# Predictors of Health Satisfaction in Spanish Physically Active Older Adults: A Cross-Sectional Observational Study

**DOI:** 10.3390/geriatrics8010027

**Published:** 2023-02-18

**Authors:** Ana Isabel Agustí, Javier Guillem-Saiz, Jesús González-Moreno, María Cantero-García, Igor Cigarroa, María Antonia Parra-Rizo

**Affiliations:** 1 Faculty of Health Sciences, Valencian International University (VIU), 46002 Valencia, Spain; 2 Department of Health Psychology, Universidad Pontificia de Comillas Madrid, 28015 Madrid, Spain; 3 Escuela de Kinesiología, Facultad de Salud, Universidad Santo Tomás, Los Ángeles 4440000, Chile; 4 Department of Health Psychology, Faculty of Social and Health Sciences, Campus of Elche, Miguel Hernandez University (UMH), 03202 Elche, Spain

**Keywords:** elderly, aging, physical activity, health satisfaction, functional ability

## Abstract

Studies that analyze the predictors of satisfaction with the health of the elderly are scarce. That is the reason why the objective of this study is to analyze whether the physical-psychological state, sports practice, and the use of socio-health resources are factors that predict satisfaction with health status in physically active elderly people. The Physical Activity and Quality of Life questionnaires were applied to a sample of 397 elderly people in this cross-sectional observational study. The data have been analyzed using Student’s *t*-test chi-square test, Cohen’s d, Phi Coefficient and Cramer’s V. The results have shown that the lack of physical illnesses (*OR* = 3.920; *p* < 0.001) and psychological problems (*OR* = 1.940; *p* = 0.032), practicing a high level of physical activity (*OR* = 2.049; *p* = 0.001), having high scores in functional skills (*OR* = 8.059; *p* < 0.001) and using little social and health services (*OR* = 2.595; *p* < 0.001) are all predictors of being highly satisfied with one’s health. In conclusion, predictors associated with high health satisfaction of active older people have been found, such as functional abilities, the existence of physical illness, psychological problems, level of physical activity, frequency of use of health and social services and satisfaction with health and social services; but it is not associated with gender or age of participants.

## 1. Introduction

The aging of the population in Spain is a reality due to the low birth rate and the increase in life expectancy, which generates a greater representativeness of the groups of people over 65 and 80 years of age, respectively [1]. In fact, according to the World Health Organization [2], prospects indicate that 30% of the Spanish population will be over 60 years of age between 2020 and 2030.

Faced with such a perspective, aging but with quality of life is important, with the term “active aging” becoming increasingly relevant. The concept of “active aging” encompasses both the personal and social spheres [3]. Therefore, there are numerous variables involved in active aging and not all of them are controllable. However, the adaptation of the person to the life cycle is a controllable variable. This adaptation process is called “active aging”.

One of the variables related to the improvement of the quality of life in the elderly is the practice of physical activity. Consequently, some authors [4,5] believe that physical activity must be considered in intervention programs to improve the quality of life of the elderly as it also influences perceptions of good health. Similarly, other authors [6] have pointed out that quality of life is linked to active and healthy aging based on physical activity, and in some research [7,8], it has been highlighted that the components that most influence the quality of life are related to health, social relations, and staying active. This last statement implies that physical activity is one of the most important strategies for disease prevention and health promotion on a physical and cognitive scale. Different studies show the benefits that the practice of physical activity has on the physical and mental health of the elderly: improvement of type II diabetes [9], cardiovascular level [10], mental health [11], and depression [12].

However, practicing physical activity is not the only factor that influences the satisfaction with life of the elderly; it is also the care of physical and mental health, among other aspects. In general, people with chronic diseases feel less satisfied with life. In this regard, some researchers [13] studied the relationships between the degree of physical activity, limitations due to disability, self-efficacy, and the quality of life in older adults and observed that an increase in physical activity was related to an increase in satisfaction with life, with physical self-efficacy and with fewer limitations derived from disability. Likewise, other academics [14] pointed out that older people with type II diabetes mellitus had a low perception of satisfaction with their health, which shows the importance of working on this aspect from different social areas. In addition, it has been seen that a low mood is related to a low ability to carry out basic activities of daily living such as bathing, dressing, or shopping [15].

In some studies [16], it has also been highlighted that comprehensive physical activity programs in older adults help increase the perception of their mental health.

However, despite the evidence that physical, mental, and social activities are key issues for healthy aging, few studies investigate these variables in relation to the improvement of health satisfaction in older adults.

Another matter addressed by this research, the autonomy of older adults and functional skills, is also closely related to the amount of physical activity that is performed, which affects the quality of life [17]. In fact, certain authors [18] emphasized the importance of promoting and improving perceived functional capacities in the elderly population. However, once again, academic papers on functional skills against dependency in relation to satisfaction with health in the physically active elderly population are scarce. For example, it has been studied the proprioceptive system, and its relationship with motor control and an association between poor mobility and frailty has been found [19], as well as an increased risk of falling [20], without relating it to aspects such as its influence on health satisfaction.

Similarly, the frequency of social and health services in relation to satisfaction with health has been barely investigated, although it is known that the frequency of use of these services among older adults helps increase their life expectancy. Some authors [21] indicated that there is an increase in the use of emergency departments as age increases, which, on the other hand, is something expected.

In this scenario, all the works related to aging and dependency are of special interest because the analysis of the use of health services by older people is essential to optimize resources [22].

Besides, old age is currently understood as a stage of life in which it is necessary for the person to maintain their vital areas at an optimal level, as opposed to the historical view of old age as a stage of loss in the physical area, cognitive and social [23]. Therefore, preventive programs that are developed for older adults could improve their quality of life [24].

However, despite the exposed scientific literature, the studies carried out in this area have not collected enough information about the elderly population that is physically active because most of them have samples of elderly people who do not practice physical exercise or who are sedentary. These investigations have not used a predictive model of health satisfaction, nor have they evaluated the impact of physical or psychological problems or the use of free health care on health. For this reason, we have studied the predictors of good health satisfaction in active older people, who have been little studied, given that the literature focuses more on people with cognitive impairment or pathologies, in order to establish a profile of active older people who age healthily, and which can be used at a local level by centers to establish preventive health policies and methods. Therefore, this publication aims to provide a predictive model of better satisfaction with health in older adults based on certain variables. Specifically, the objective of this study is to analyze whether the physical-psychological state, sports practice and the use of socio-health resources are factors that predict satisfaction with health status in physically active elderly people.

## 2. Materials and Methods

### 2.1. Participants

A cross-sectional observational was carried out. Initially, 613 participants were enrolled in the study and received the questionnaires. There were 89 participants who never returned their completed questionnaires. After review of the completed questionnaires, 127 participants were discarded because their copies contained major inconsistencies, had unanswered items, or it was advisable after the result obtained from the exploratory statistical analysis of the data.

Finally, 397 older people (64.7% were women and 35.3% were men) with a mean age of 69.65 years (SD = 4.71) were included to participate. Sampling was non-probabilistic by convenience.

Participants were included on the basis of (a) people over 60 years of age, (b) older people attending social and/or sports centers for at least one year, and (c) older people who could answer the questionnaires. On the other hand, the exclusion criteria were (a) sedentary people (it was indicated in the instructions to have at least one year of sports practice to participate in the study), (b) people who had difficulties in reading the questionnaire battery, and (c) people who did not participate in the physical or sporting activities. They were surveyed in their sports and social centers after their sports practice. They could even return the questionnaires at a later appointment.

Participation was voluntary and anonymous. The confidentiality of the data has been guaranteed in accordance with the General Data Protection Regulation, as well as the Organic Law 3/2018, of 5 December, on Personal Data Protection and Guarantee of Digital Rights, and this study was approved by the Ethics Committee of the Miguel Hernández University (registration number 200115191342).

### 2.2. Procedure

A cross-sectional observational study has been planned. The type of sampling was convenience sampling. Participants were selected in two settings in Alicante: in sports and social centers and in outdoor spaces where the sport is usually practiced.

On the one hand, 38 centers were contacted, of which 18 agreed to collaborate. Those interested in participating were given a copy of the informed consent form and another copy of the questionnaire, which they completed individually after the physical activity. 

On the other hand, in external areas where the population goes to practice sports, contact was made with people who met the requirements defined for the study, and the research and its objectives were explained to them. Those who agreed to participate were given an envelope with the questionnaire and the informed consent form, which they had to fill in and return at a later appointment set at that time. Those interested, and after the informative meetings in which the objective of the research was explained, were given the informed consent form and the rest of the questionnaire battery, which they had to hand in afterward. 

### 2.3. Variables and Measured Instruments

#### 2.3.1. Physical Activity

This outcome was measured with the International Physical Activity Questionnaire, IPAQ [25]. This questionnaire shows three levels of activity: high, moderate and low < 3 METs, 3–6 METs and >6 METs, respectively [26]. Performing a moderate-intensity level or half an hour of vigorous-intensity level corresponds to a high level of physical activity. Performing at least half an hour of moderate-intensity physical activity almost every day corresponds to a moderate level of activity. Finally, when people are not at moderate or high levels, it corresponds to a low level of activity [27]. The reliability of the IPAQ short version is 0.65 (rs = 0.76; CI 95%: 0.73–0.77). This test is currently used in the elderly population [28].

#### 2.3.2. Quality of Life

The questionnaire used to assess the quality of life was the Brief Quality of Life Questionnaire [29]. For this study, from the quality of life questionnaire, we used functional abilities, health and social services, and physical and mental health. It is a highly recommended questionnaire for assessing participants’ quality of life [29]. The participant evaluates 71 items that assess the degree of satisfaction or the frequency of different questions such as “FREQUENCY with which you use the social and health services offered by the community or residence” in a response of “frequently, occasionally or never”. The internal consistency of the scale ranges between 0.70 and 0.92. This questionnaire is currently used in the elderly population [30].

#### 2.3.3. Sociodemographic Variables

A questionnaire related to sociodemographic data was drawn up that collected the sex, age, marital status, habits and health status, cohabitation situation and employment status and income of the participants.

### 2.4. Analysis of Data

Firstly, the data were analyzed beforehand, and a descriptive analysis of the variables according to the level of satisfaction was carried out using means and standard deviations for quantitative variables and frequencies for qualitative variables. The distribution of data was analyzed through a Kolmogorov–Smirnoff test, and the equality of variances through a Levene test, which showed a normal distribution and homogeneity of variances, respectively.

An independence analysis of the study variables was performed by applying the Student’s *t*-test for independent samples in the continuous variables and the chi-square test in the categorical variables. The effect size was estimated using Cohen’s d for quantitative variables and the Phi Coefficient and Cramer’s V for qualitative variables (depending on the size of the contingency tables).

Subsequently, a logistic regression analysis was carried out using the Enter method with a view to studying whether the variables analyzed allowed for predicting the level of satisfaction with the health of the physically active elderly. The data analysis was carried out with the statistical package SPSS 25 (SPSS Inc., Chicago, IL, USA), with a significance level of *p* < 0.05. 

## 3. Results

The results of the study show that 7.1% of participants said that they were not at all satisfied with their state of health, 25.2% were somewhat satisfied, 50.1% were quite satisfied, and 17.6% were very satisfied with their health. Based on these data, the subjective health variable, which reflects satisfaction with the state of health, has been dichotomized into two groups: 0 = low satisfaction with health and 1 = high satisfaction with health.

The relationships between the level of satisfaction, the health status of the participants, and the study variables were analyzed. The analysis shown in Table 1 indicates that satisfaction with the health of the elderly is related to the scores they obtain in functional skills (t (184.086) = 7.358; *p* < 0.001; d = 0.89).

Table 2 shows that satisfaction with the health status of the elderly is also related to the existence of physical illnesses (χ^2^(1, N = 397) = 35.896; *p* < 0.001; Phi = 0.301), with the presence of psychological problems (χ^2^(1, N = 397) = 4.617; *p* = 0.032; Phi = 0.108), with the level of physical activity (χ^2^(2, N = 397) = 11.371; *p* = 0.003; VCramer = 0.169), with the frequency of use of social and health services (χ^2^(2, N = 397) = 19.281; *p* < 0.001; VCramer = 0.220), and with satisfaction with social and health services (χ^2^(3, N = 397) = 16.930, *p* = 0.001, VCramer = 0.207). However, it is not associated with gender (χ^2^(1, N = 397) = 0.001; *p* = 0.975; Phi = 0.002), nor with the age of the participants (χ^2^(1, N = 397) = 0.611; *p* = 0.435; VCramer = 0.039).

In order to study which of the variables have provided statistical significance in the association analyses are predictive of satisfaction with the state of health of physically active older adults, the continuous variable of social skills has been dichotomized by dividing the participants into two groups (0 and 1) according to the median: 203 elderly (51.1%) formed group 0 and presented high levels of functional abilities (≥4.00), and 194 elderly (48.9%) formed group 1 and presented low levels of functional abilities (<4.00). Likewise, in the qualitative variables, the level of physical activity variable has been dichotomized, grouping the 183 participants who practice high physical activity in group 0 (46.1%), the 214 who practice low or moderate physical activity in group 1 (53.9%), and the variable frequency of use of social and health services grouping the 250 participants who never or occasionally use social and health services in group 0 (63%) and the 147 who use them frequently in group 1 (37%).

**Figure 1:**
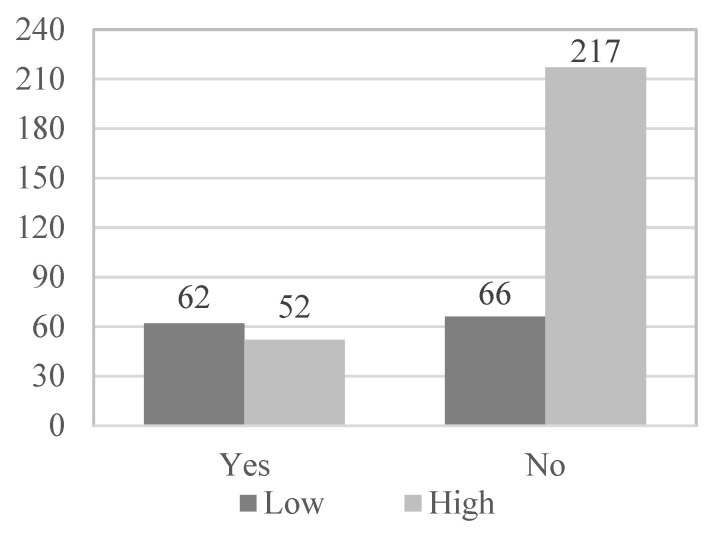
Physical illness_Health satisfaction.

**Figure 2:**
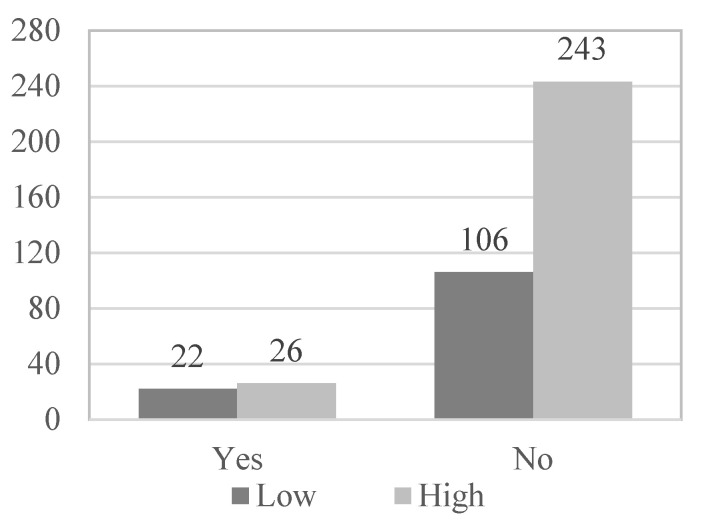
Psychological problem_Health satisfaction.

**Figure 3:**
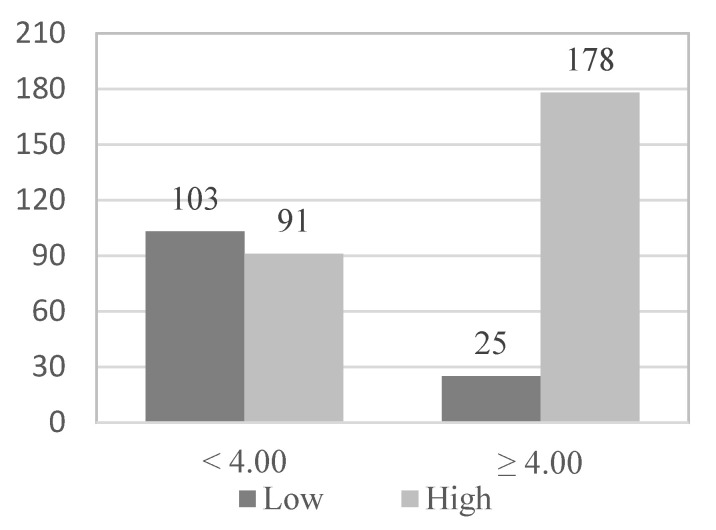
Functional skills_Health satisfaction.

**Figure 4:**
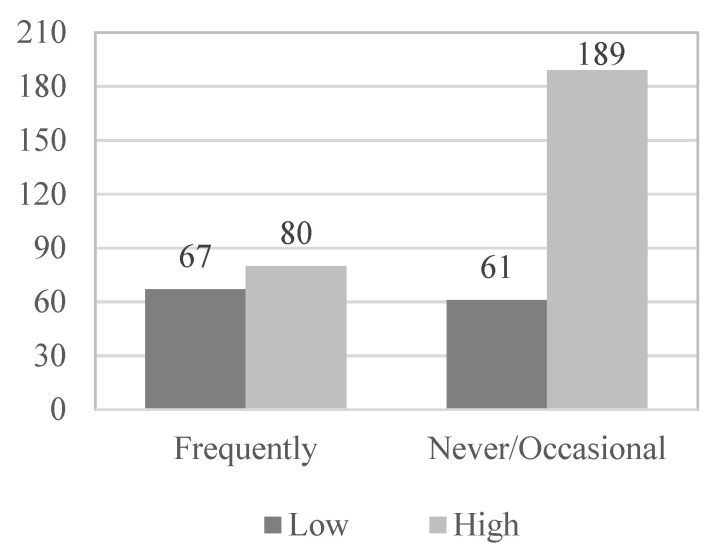
Use of social and health services_Health satisfaction.

**Figure 5:**
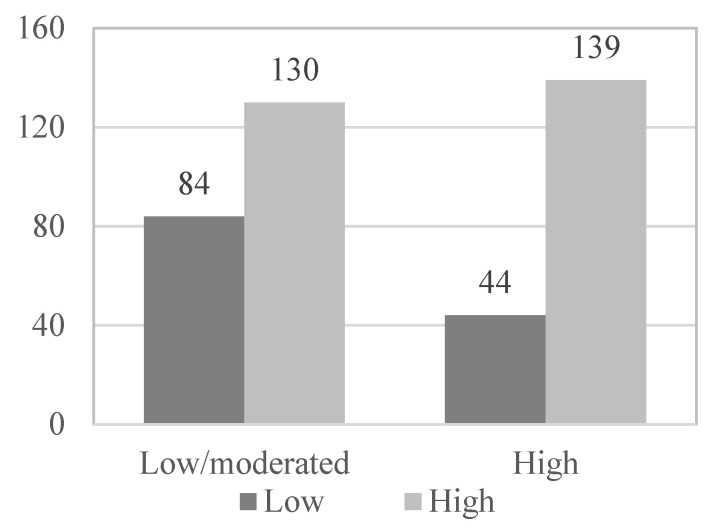
Physical activity level_Health satisfaction.

Subsequently, in the multivariate logistic regression model, those variables that have obtained statistical significance in the bivariate analysis and provide a more harmonious model have been introduced as variables: the existence of physical illness (OR = 3.920; *p* < 0.001), presence of psychological problems (OR = 1.940; *p* = 0.032), functional abilities (OR = 8.059; *p* < 0.001), use of social and health services (OR = 2.595; *p* < 0.001) and physical activity level (OR = 2.049; *p* = 0. 001) (Figure 1, Figure 2, Figure 3, Figure 4 and Figure 5).

It has been found that the most illustrative variable of having a high level of satisfaction with the state of health is the functional abilities variable (7.3 times higher for the elderly with a high level of functional abilities) (Table 3).

## 4. Discussion

### 4.1. Main Findings of This Study

The satisfaction with the health of the elderly is related to the scores they obtain in functional skills. Satisfaction with the health status of the elderly is also related to the existence of physical illnesses, the presence of psychological problems, the level of physical activity, the frequency of use of social and health services, and satisfaction with social and health services. However, it is not associated with gender or with the age of the participants. In addition, the most illustrative variable of having a high level of satisfaction with the state of health is the functional abilities variable.

### 4.2. How Can the Results Be Interpreted in the Perspective of Previous Studies?

The model shows that the fit of the data is good (Hosmer and Lemeshow test > 0.05). The proportion of variance explained by the model of level of satisfaction with health status is between 25.4% (Cox and Snell’s R2) and 35.5% (Nagelkerke’s R2). The model allows the correct estimation of 75.6% of the cases, with the sensitivity of the model being 85.5% and the specificity being 54.7%.

The aim of this publication was to analyze whether the physical and psychological state of physically active older adults, the intensity of their sports practice and the use of social and healthcare resources are factors that can predict satisfaction with their state of health.

Our results indicate that physical health status, psychological status, the practice of physical activity and the use of socio-health services are all predictors of satisfaction with health. Also, a relationship between physical activity and satisfaction with health has been found. In this regard, some authors [31] pointed out that the practice of a physical activity is an indicator of the maintenance of factors related to health satisfaction in people over 60 years of age. In fact, high levels of physical activity daily can favor a state of regular mental health, providing the person with tools to deal with the problems associated with old age [32].

The relationship between physical activity and perceived health in the elderly is favorable when the physiological changes derived from its practice are correlated with aspects such as self-care and personal satisfaction in the participants of the study. On the other hand, significant results have been obtained in some variables of the health questionnaire in those participants who showed greater adherence to exercise and more changes in cardiorespiratory capacity [33]. Moreover, there have been studies with favorable results in those subjects who performed a physical activity of greater intensity [34]. Consequently, it could be concluded that physical activity is useful in preventing the development of physical illnesses, and, in addition, it is therapeutic as it improves the parameters of the quality of life and health of the elderly [35].

Other authors [36] maintain that longevity must be accompanied by a good quality of life, a matter that is closely related to a healthy lifestyle (diet, physical exercise, cognitive and intellectual level, interpersonal relationships) and, quite the reverse with issues such as sedentary lifestyle, and the consumption of tobacco, drugs, and alcohol. In this way, the physical problems of the older adult cause their satisfaction with health to be reduced as well as their perception of their health status to decrease.

The results of this study also show relationships between older adults’ physical problems and feeling more satisfied with their health. In this regard, similar studies find a relationship between a good psychological state and the practice of physical exercise as a protective factor against physical health problems [37,38].

In relation to the existing literature in the area and in line with the results of this study, satisfaction in the elderly is predicted when they achieve favorable results in functional skills [39] for carrying out activities of daily living such as taking care of one’s own appearance, getting dressed and performing domestic tasks (or outside the home), which in turn means that the older adult has a good assessment of satisfaction with health.

With reference to socio-health services, it should be noted that certain studies [40] confirm, like this research, that practicing some type of physical activity encourages older adults to be active, to feel satisfied with their health and, consequently, to lower their health expenditure. Regarding the latter, it should be noted that no studies have been found that support the results obtained in this research, so this study represents an interesting contribution to the scientific literature: assistance of the health sector to older adults helps them feel more satisfied with their own health.

Finally, all the changes and challenges in the world today, including climate change, depletion of natural resources, COVID-19, and wars, must be taken into account as consequences that affect psychological well-being and could have a great impact on mental health in the 21st century [41], so there are multiple variables that affect well-being and satisfaction and even mental health today.

### 4.3. Limitations

However, this research has some limitations linked to the cross-sectional study and the impossibility of extrapolating the results to the general older adult population, although its uniqueness should also be highlighted due to the lack of studies that provide a predictive model of the variables that improve satisfaction with health in physically active older adults. In addition, since the questionnaires were self-administered and subject to the participant’s perception, the sample size is small and not representative of the population. Moreover, considering that the health satisfaction variable is multifactorial and complex, only a few factors that can modulate it were measured.

### 4.4. Contributions and Practical Implications

In terms of scientific impact or theoretical implications:

The study will help to reinforce the existing incipient scientific evidence on the physical, psychological and social predictors of health satisfaction in active older people.

In this context, the results emanating from this project would become the most up-to-date local evidence on physical, psychological and social variables that modulate health satisfaction in active older people.

In the same vein, this project can help to make visible within the scientific community the issue related to the generation of a predictor model of health satisfaction of the elderly.

In addition, the results of this project may encourage the community of health and social professionals who work daily with older people to search for and identify physical, psychological, and social traits or characteristics that may help to identify older people who feel good about themselves or who are aging healthily.

On the other hand, this work opens a line of research little explored in Spain related to predictive models of health satisfaction in older people and understanding the variables that can predict health satisfaction in older adults. In fact, recent multidomain research on cognitive training, physical activity, and nutritional advice supports the benefits of these issues on health satisfaction [42], which suggests that the adoption of preventive measures can improve the autonomous capacity of older adults.

In terms of practical implications:

These results can serve as evidence so that decision-makers in community, social or sports centers can generate plans or programs to investigate predictors of health satisfaction. On the other hand, strategies could be proposed to promote healthy aging based on the promotion of variables that were detected as predictors of low health satisfaction.

Concretely, the methodology of the project could be replicated in other social or sports centers and thus confirm these results in other populations of older people, in other environments with different barriers and facilitators of health satisfaction, and improvement in prevention and intervention in the variables that have been significant in this study can be beneficial for the enhancement of the final quality of life as well as the reduction of dependency [43].

### 4.5. Future Line of Research

Considering the present results, a future line of research could extend the sample to the sedentary, inactive, frail, frail, elderly population with comorbidities and polypharmacy, among others.

Extending the study variables that could be predictors of health satisfaction, such as mental health variables, variables associated with drug consumption, variables associated with sexual activity or sleep quality, and finding out what other variables not taken into account in this study can cause a greater positive effect in relation to satisfaction with health. It would also be advisable to improve the evaluation of physical activity through biometric measurement methods and not only through a self-administered questionnaire.

## 5. Conclusions

This research shows that the existence of physical illnesses, the presence of psychological problems, the level of physical activity practiced, functional skills and the frequency of use of socio-health services are predictive variables of the level of satisfaction with the state of health in the active seniors. We have found that the level of physical activity practiced, frequency of use of social and health services, and physical-emotional state (described as the functional abilities available to them, the existence of physical illnesses, and the presence of psychological problems) are predictors of the level of satisfaction with their state of health in active older people.

Consequently, predictors associated with high health satisfaction of active older people have been found. These results could help in the construction of a physical, psychological and social profile of the older population with high satisfaction and, consequently, with a high probability of healthy aging.

## Figures and Tables

**Table 1:** Means, standard deviations and *t*-tests of the scores on the functional abilities scale according to the level of satisfaction with health.

	Satisfaction with Health					
Scale	High		Low		t	*p*
	*n*	M (SD)	*n*	M (SD)		
Functional skills	269	3.80 (0.34)	128	3.45 (0.50)	7.358	0.000 *

*Note.* * *p* < 0.05; *n* = number of participants, *M*(*SD*) = mean and standard deviation, *t* = statistical *t*-student, and *p* = *p*-value.

**Table 2:** Frequencies of genders, age, physical illnesses, psychological problems, PA level and use and satisfaction of socio-health services according to the subjective health of the participants.

	*Satisfaction with Health*					
	High		Low		Total	
	*n*	%	*n*	%	*n*	%
*Gender*						
Male	95	35.3	45	35.2	140	35.3
Female	174	64.7	83	64.8	257	64.7
*Age*						
<70 years old	185	68.8	83	64.8	268	67.5
≥70 years old	84	31.2	45	35.2	129	32.5
*Physical illnesses*						
Yes	52	19.3	62	48.4	114	28.7
No	217	80.7	66	51.6	283	71.3
*Psychological problems*						
Yes	26	9.7	22	17.2	48	12.1
No	243	90.3	106	82.8	349	87.9
*Physical activity*						
Low	27	10.0	22	17.2	49	12.3
Moderate	103	38.3	62	48.4	165	41.6
High	139	51.7	44	34.4	183	46.1
*Use of socio-health services*						
Never	17	6.3	7	5.5	24	6.1
Occasionally	172	63.9	54	42.2	226	56.9
Frequently	80	29.8	67	52.3	147	37.0
*Satisfaction with socio-health services*						
Wholly dissatisfied	5	1.9	7	5.5	12	3.0
Moderately dissatisfied	52	19.3	33	25.8	85	21.4
Moderately satisfied	161	59.9	50	39.1	211	53.2
Very satisfied	51	18.9	38	29.6	89	22.4

*Note. n* = number of participants; % = percentage.

**Table 3:** Logistic regression model of the level of satisfaction with health.

	B	E.T.	Wald	*p*	Exp(B)	IC 95 for Exp(B)
*Physical illness*	1.174	0.269	19.004	0.000 *	3.234	1.908–5.481
*Psychological problem*	0.723	0.368	3.861	0.049 *	2.061	1.002–4.241
*Functional skills*	1.983	0.276	51.763	0.000 *	7.263	4.232–12.466
*Use of social and health services*	0.578	0.257	5.045	0.025 *	1.783	1.076–2.952
*Level P.A.*	0.380	0.262	2.112	0.146	1.463	0.876–2.442
*Constant*	−1.991	0.433	21.140	0.000 *	0.136	

*Note.* * *p* < 0.05; *B* = Coefficient b; *S.E.* = standard error; *Wald* = Wald statistic; *p* = *p*-value; *Exp*(*B*) = Odds Ratio; *IC95 for Exp(B)* = confidence interval 95% Odds Ratio.

## Data Availability

The database is under the custody of the IP with anonymized data.
